# Brain Metabolite Diffusion from Ultra-Short to Ultra-Long Time Scales: What Do We Learn, Where Should We Go?

**DOI:** 10.3389/fnins.2018.00002

**Published:** 2018-01-19

**Authors:** Julien Valette, Clémence Ligneul, Charlotte Marchadour, Chloé Najac, Marco Palombo

**Affiliations:** ^1^Commissariat à l'Energie Atomique et aux Energies Alternatives, MIRCen, Fontenay-aux-Roses, France; ^2^Neurodegenerative Diseases Laboratory, Centre National de la Recherche Scientifique, Université Paris-Sud, Université Paris-Saclay, UMR 9199, Fontenay-aux-Roses, France; ^3^Department of Computer Science and Centre for Medical Image Computing, University College of London, London, United Kingdom

**Keywords:** intracellular diffusion, brain metabolites, ADC time-dependency, microstructure, diffusion time

## Abstract

*In vivo* diffusion-weighted MR spectroscopy (DW-MRS) allows measuring diffusion properties of brain metabolites. Unlike water, most metabolites are confined within cells. Hence, their diffusion is expected to purely reflect intracellular properties, opening unique possibilities to use metabolites as specific probes to explore cellular organization and structure. However, interpretation and modeling of DW-MRS, and more generally of intracellular diffusion, remains difficult. In this perspective paper, we will focus on the study of the time-dependency of brain metabolite apparent diffusion coefficient (ADC). We will see how measuring ADC over several orders of magnitude of diffusion times, from less than 1 ms to more than 1 s, allows clarifying our understanding of brain metabolite diffusion, by firmly establishing that metabolites are neither massively transported by active mechanisms nor massively confined in subcellular compartments or cell bodies. Metabolites appear to be instead diffusing in long fibers typical of neurons and glial cells such as astrocytes. Furthermore, we will evoke modeling of ADC time-dependency to evaluate the effect of, and possibly quantify, some structural parameters at various spatial scales, departing from a simple model of hollow cylinders and introducing additional complexity, either short-ranged (such as dendritic spines) or long-ranged (such as cellular fibers ramification). Finally, we will discuss the experimental feasibility and expected benefits of extending the range of diffusion times toward even shorter and longer values.

## Introduction

While water molecules are ubiquitous in the brain, many metabolites detected by magnetic resonance spectroscopy (MRS) *in vivo* are primarily intracellular, with typical extracellular concentrations ~1,000–10,000 times lower than intracellular concentrations. Moreover, works on extracts or cell cultures suggested that some metabolites exhibit preferential compartmentation in different cell types, with glutamate (Glu) and N-acetylaspartate (NAA) predominantly found in neurons, and myo-inositol (Ins) and choline compounds (tCho) preferentially found in glial cells (Simmons et al., 1991; Brand et al., 1993; Griffin et al., 2002; Le Belle et al., 2002; Choi et al., 2007) (in particular astrocytes, representing the largest volume fraction of glial cells). Hence, measuring the brain metabolite diffusion may provide specific insight into cellular organization and microstructure.

The intracellular compartmentation of metabolites may *a priori* seem to simplify interpretation and modeling of metabolite diffusion as compared to water, because extracellular space and membrane permeability may be neglected. However, it is conceivable that metabolites are highly compartmentalized at a subcellular scale, in some subcellular regions or organelles. For example, NAA has been reported to be synthetized in mitochondria (Madhavarao et al., 2003), so that it may be primarily found in mitochondria. In this context, metabolite diffusion would reflect its subcellular metabolic compartmentation rather than diffusion within the whole cytosol and restriction by cell membrane. Here we will see how studying the time-dependency of metabolite apparent diffusion coefficient (ADC) allows clarifying the nature of the compartments where MRS-detected metabolites are diffusing, and may allow probing morphological features at different spatial scales. We will then try to explain some motivations and approaches to push further the limits of achievable diffusion times.

## What does ADC time-dependency tell about metabolite diffusion?

To measure metabolite ADC at very short time-scales, experiments were performed in the rat (Marchadour et al., 2012) and mouse brain (Ligneul and Valette, 2017) using oscillating gradients. Measurements frequencies *f* went up to ~250 Hz, corresponding to diffusion time *t*_*d*_ down to ~0.5–1 ms, depending on the conversion used between *f* and *t*_*d*_ [*t*_*d*_ = 1/(4*f*) based on the identification of the effective diffusion time in the *b*-value expression (Parsons et al., 2006), or using *t*_*d*_ = 9/(64*f*) as derived in the Mitra limit when considering the surface-to-volume ratio of the restrictions (Novikov and Kiselev, 2011)]. They showed that ADC increased by ~50% when *f* increased from ~20 to 250 Hz for NAA, tCho, and tCr (also for Ins and Tau in the mouse brain), approaching ADC ~0.2–0.30 μm^2^/ms at the highest frequency. Note that, although later measurements in the mouse brain (Ligneul and Valette, 2017) suggested that early measurements in rats (Marchadour et al., 2012) may have been slightly biased by some motion artifact for some frequencies, the overall trend was preserved. The large ADC increase at short time-scales reflects significantly decreased restriction/hindrance and the progressive approach toward free diffusion. It rules out any significant contribution of active transport at these scales, which would result in the opposite trend (as the velocity autocorrelation function would be positive and decrease to 0 for increasing time Does et al., 2003). At ~1 ms, the typical diffusion distance for metabolites is ~1 μm, so the typical distance between obstacles/walls inducing ADC time-dependency must be in this range. More quantitatively, modeling metabolite ADC acquired in the rodent brain with oscillating gradients (Marchadour et al., 2012; Ligneul and Valette, 2017) using Stepisnik's and Callaghan's frequency-domain formalism for diffusion in cylinders or spherical pores (Stepisnik, 1981; Callaghan and Stepisnik, 1995) yields typical radii of ~1 μm. It also allows estimating the free intracellular diffusivities to be *D*_*intra*_ ~0.5–0.6 μm^2^/ms, i.e., corresponding to a low-viscosity cytosol, less than twice the viscosity of pure water). This is in excellent agreement with fluorescence-based estimates of fluid-phase cytoplasm viscosity being quite similar to bulk water (Fushimi and Verkman, 1991; Luby-Phelps et al., 1993).

**Figure 1:**
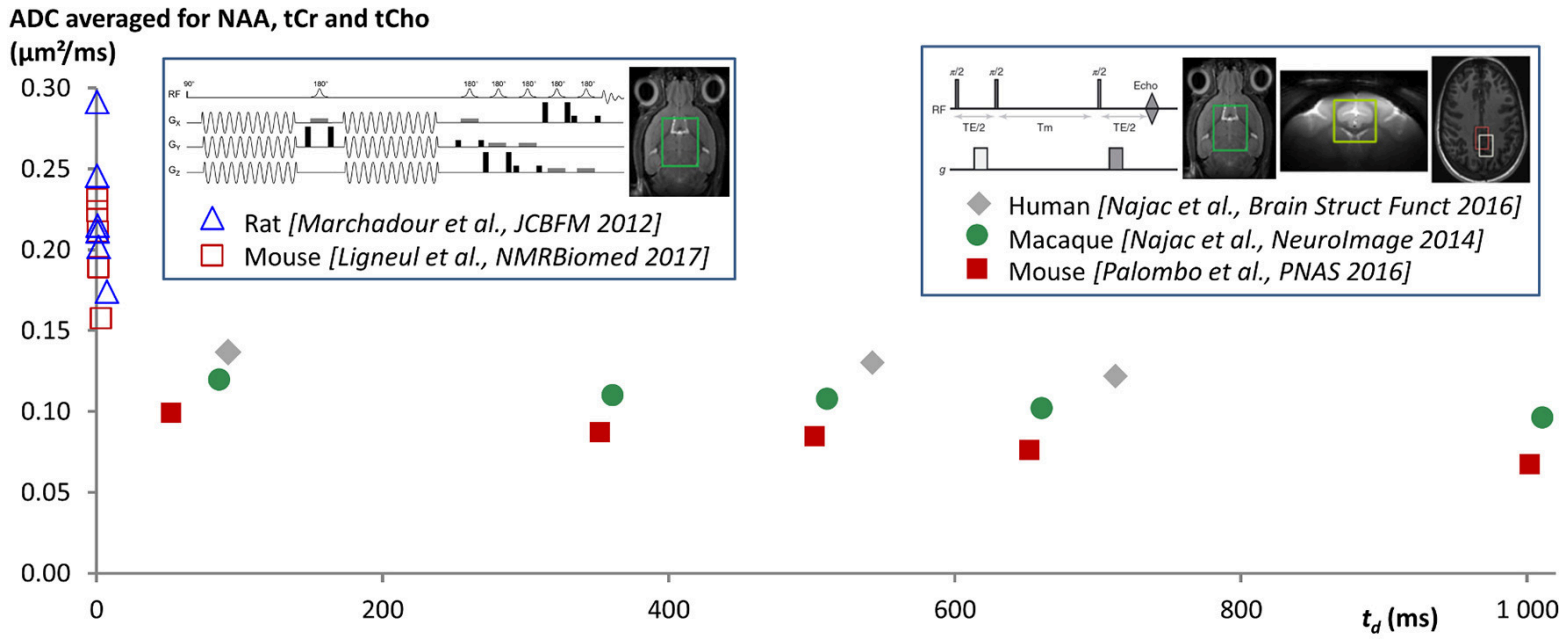
ADC time-dependency as measured in different published works from ultra-short *t*_*d*_ using oscillating gradients [down to *t*_*d*_ ~ 0.5 ms when taking *t*_*d*_ = 9/(64*f*) (Novikov and Kiselev, 2011), where *f* is the oscillating gradient frequency], up to very long *t*_*d*_ ~ 1 s using stimulated echo (data acquired at *t*_*d*_ ~ 2 s and reported in Palombo et al., 2016a are not shown here for clarity). Data points are ADC averaged for NAA, tCho, and tCr, but the trend is similar for each metabolite. The fact that ADC drops when *t*_*d*_ is increased at very short *t*_*d*_, and then remains fairly stable at approximately one third of the ADC value measured at ultra-short times, is very consistent with metabolite diffusion mainly occurring in long and thin fibers (indeed, in the ideal situation of infinitely long and straight fibers, and in the case of an isotropic distribution of fiber orientations, ADC would drop from *D*_*intra*_ at infinitely short *t*_*d*_ to *D*_*intra*_/3 once full restriction has been reached in the plane perpendicular to fiber axis, and then would stabilize at *D*_*intra*_/3 at longer *t*_*d*_, where *D*_*intra*_ is the free intracellular diffusivity). In a situation with metabolites massively confined in subcellular regions such as organelles or cell bodies, the ADC would drop to ~ 0 at long *t*_*d*_ over the observed time-range. Active transports would rather lead to ADC increasing with *t*_*d*_, at least for the time-scales at which these transports become significant compared to diffusion.

Measurements at longer *t*_*d*_ were achieved using pulsed-field gradients. Most DW-MRS works investigating brain metabolite diffusion were performed at a single *t*_*d*_, in the 10–250 ms range, and reported ADC in the 0.1–0.25 μm^2^/ms range (see for example Merboldt et al., 1993; Wick et al., 1995; Dreher et al., 2001 for measurements performed at *t*_*d*_ slightly longer than 10 ms, and Posse et al., 1993; Ellegood et al., 2006 for measurements at *t*_*d*_ > 200 ms). We are not aware of many works investigating *t*_*d*_-dependency, except pioneer works where the high *b*-value attenuation of NAA was studied in the 35–305 ms range in excised rat brains (Assaf and Cohen, 1998a,b) and in the 50–100 ms range in the living rat brain (Kroenke et al., 2004). However, because acquisition and analysis differed between these studies, and some of them may be prone to artifacts (e.g., absence of scan-to-scan phase correction, incorrect *b*-value calculation ignoring cross-terms, signal contamination by macromolecules…), it is very difficult to see any clear pattern emerge in terms of ADC time-dependency. To specifically study ADC time-dependency at long *t*_*d*_, a series of studies were performed in the rodent, primate and Human brain, all based on stimulated echo acquisitions (which are more favorable than spin echo to reach long *t*_*d*_, as magnetization relaxes according to T1 during the mixing/diffusion time) and designed to minimize measurement bias (Najac et al., 2014, 2016; Palombo et al., 2016a). These studies all report very stable ADC (around 0.1 μm^2^/ms, except in Human white matter where it was ~0.15 μm^2^/ms) for all metabolites as *t*_*d*_ is increased from a few dozen ms up to ~700 ms in Human brain (Najac et al., 2016), and up to ~2 s in the mouse and macaque brain (Najac et al., 2014; Palombo et al., 2016a). Note however that a slight trend to decrease can be observed (at least in the mouse and primate brain) when considering the whole time-window. The fact that ADC does not drop as *t*_*d*_ is increased up to 2 s is a very clear indication that metabolites are not confined in closed compartments of size equivalent to the diffusion distance (30–40 μm) or below, such as cell bodies or organelles (e.g., see Figure 3 in Najac et al., 2016). Instead, the fact that ADC remains fairly stable at approximately one third of the ADC value measured at ultra-short time-scales (Figure 1) is very consistent with metabolite diffusing in long fibers (in the ideal situation of infinitely long and straight fibers, and in the case of an isotropic distribution of fiber orientations, ADC would drop from *D*_*intra*_ at *t*_*d*_ ~ 0 to *D*_*intra*_/3 once full restriction has been reached in the plane perpendicular to fiber axis, and then stabilize at *D*_*intra*_/3 at longer *t*_*d*_).

This framework of diffusion in long fibers can obviously only be considered as an approximation. First, cells do not consist of hollow tubes with smooth surfaces. The cytosol is filled with cytoskeleton and organelles which might induce tortuous diffusion. Similarly, dendritic spines and astrocytic leaflets, which are small protrusions along the dendrites and astrocytic processes, will slow down metabolites longitudinal diffusion, also resulting in tortuous diffusion along fiber axis. These features will result in decreased ADC plateauing at *D*_*intra*_/τ along fibers at long *t*_*d*_ or, in the case of an isotropic distribution of fiber orientations or an isotropic ADC measurement (trace of the tensor), at *D*_*intra*_/(3τ), where τ is the tortuosity along fibers. Theoretical considerations predict that this asymptotic value should be approached according to a −1/2 power-law in the case of a random distribution of obstacles with short-range disorder, i.e., ADC(*t*_*d*_) ~ ADC(*t*_*d*_ = ∞) + K*t*_*d*_^−1/2^, where K depends on the correlation length of the obstacles (Novikov et al., 2014). However, it is not trivial to determine when the tortuosity limit would be reached in practice, i.e., at what *t*_*d*_ would the ADC approach this asymptotic value within a few percent, which can be considered as a practical threshold due to limited measurement precision. Works based on numerical simulations suggested that tortuosity limit imposed by spines or similar structures with realistic densities (i.e., in the range of 0–5 μm^−1^) would be reached within ~200 ms (Santamaria et al., 2006; Palombo et al., 2017), corresponding to a typical diffusion distance of ~10 μm along fibers. Hence the ADC value at ~200 ms could be a good estimate of *D*_*intra*_/(3τ), which would allow extracting the tortuosity induced by short-range structures, provided *D*_*intra*_ is known. Considering the values of *D*_*intra*_ ~0.5–0.6 μm^2^/ms estimated from modeling of oscillating gradients data in the rodent brain, and ADC ~ 0.1 μm^2^/ms at *t*_*d*_ ~ 200 ms in the rodent brain, one gets τ ~ 1.6–2, corresponding for example to 2–4 spines/leaflets per μm (see Table 1 in Palombo et al., 2017). This is however an upper estimate for τ. Indeed, other works based on the analysis of high b-value at a single *t*_*d*_, using models of diffusion in cylinders, have reported *D*_*intra*_ to be rather ~0.3–0.45 μm^2^/ms (Kroenke et al., 2004; Palombo et al., 2016b), which would rather correspond to a very low intracellular tortuosity τ ~ 1–1.3 (corresponding to 0–1 spines or leaflets per μm), but these high-b values experiments were performed at *t*_*d*_ ~ 50–60 ms, so that *D*_*intra*_ might actually already include some tortuosity. This illustrates how the estimation of τ depends on estimation of *D*_*intra*_, which remains indirect as it relies on some modeling.

Once the tortuosity limit imposed by short-range structures has been reached (beyond *t*_*d*_ ~ 200 ms), structural features at larger spatial scales (>~10 μm) are also expected to induce some temporal dependency of ADC, again challenging the approximation of diffusion in long cylinders. These structural features may include fiber undulation, fiber branching, and finite fiber length, which may explain the slight trend of ADC to decrease when increasing *t*_*d*_ up to ~2 s. The effect of undulations on intracellular diffusion has been investigated using analytical models or numerical simulations (Nilsson et al., 2012), while the effect of branching and finite length has been modeled using numerical simulations (Palombo et al., 2016a). Actually, the later approach was used to analyze experimental data, and suggested that realistic fiber branching and length were indeed quantitatively able to explain observed ADC time-dependency up to 2 s. In particular, diffusion compartments of supposedly astrocytic metabolites were found to be smaller and less complex than those of supposedly neuronal metabolites, and to be also smaller in rodents than in primates, consistently with histology (Palombo et al., 2016a). In this latter work, an intracellular diffusion coefficient including some short-range tortuosity along fibers (equivalent to *D*_*intra*_/τ) as discussed in the previous paragraph was also let as a free parameter in addition to morphometric parameters, and was found to be ~0.3–0.45 μm^2^/ms, i.e., lower than those derived from oscillating gradient data, but very close to values derived from high-b values (Kroenke et al., 2004; Ligneul et al., 2017b), suggesting that the latter also already include short-range tortuosity. It is worth mentioning that these long-range structures are of different natures: in theory, undulation and branching would result in “long-range” tortuosity toward a non-zero ADC value, while finite length would result in full restriction with a *t*_*d*_^−1^ approach toward zero. However, these different trends remain hypothetical, as they would become manifest only at very long *t*_*d*_ (several seconds or even tenths of seconds); at such *t*_*d*_ other phenomena such as intercellular trafficking, enzyme binding or biochemical transformations may become significant and obscure any long-time power law induced by structure.

## Why and how to get further?

**Figure 2:**
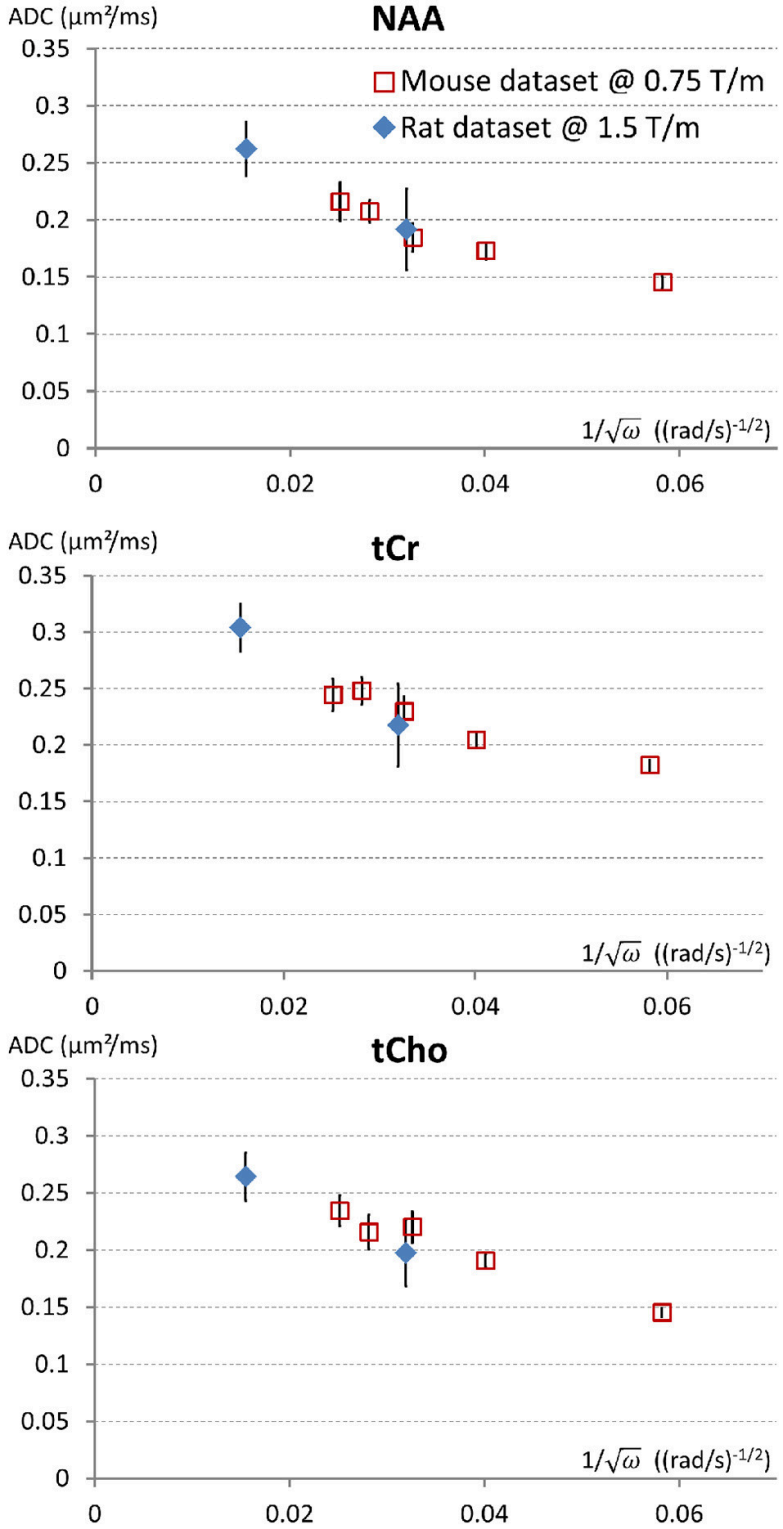
Trying to approach the free intracellular diffusion coefficient of brain metabolites using oscillating gradients. On this figure the ADC measured for NAA, tCr, and tCho, in the mouse brain at 11.7 T using a gradient coil capable of reaching 0.75 T/m along each axis (red open squares) (Ligneul and Valette, 2017) and the rat brain at 7 T using a gradient coil capable of reaching 1.5 T/m along each axis (blue diamonds) (Ligneul et al., 2017a), are displayed as a function of the inverse of square root of the angular frequency [since a linear trend is expected for ADC(ω^−1/2^) in the Mitra limit]. For better consistency between datasets, the first rat data obtained with oscillating gradients (Marchadour et al., 2012), which were probably slightly biased toward higher values due to some motion artifact, are not displayed here. Data points on the left (smallest ω^−1/2^) correspond to maximal OG frequency *f* = 665 Hz. Error bars stand for standard errors of the mean.

As discussed above, the determination of *D*_*intra*_ remains indirect and uncertain. However, it is in theory possible to directly measure *D*_*intra*_ provided ADC is measured at sufficiently short time-scales. Simulations in fibers with “realistic” spines/leaflets size and density, as performed in Palombo et al. (2017) but over an extended frequency range, suggest that oscillating gradient frequencies of at least 2,500 Hz are required to approach *D*_*intra*_ within less than 10% (assuming *D*_*intra*_ = 0.5 μm^2^/ms). Reaching such high frequencies while maintaining sufficiently high *b* (~1 ms/μm^2^ or higher) to reliably measure signal attenuation is extremely challenging. We have obtained preliminary metabolites ADC measurements up to *f* = 665 Hz in the rat brain (Ligneul et al., 2017a), using a gradient coil capable of reaching 1.5 T/m within 250 μs along each axis. This corresponds to *t*_*d*_ ~ 0.2 ms, when using *t*_*d*_ = 9/(64*f*) in the Mitra limit (Novikov and Kiselev, 2011). ADC values of 0.25–0.3 μm^2^/ms were measured, which actually well extend the trend toward higher ADC at higher frequencies as recently reported in the mouse brain (Ligneul and Valette, 2017), as shown in Figure 2. Although we are presumably far from reaching sufficiently high frequencies to directly get ADC ~ *D*_*intra*_, it is actually tempting to estimate *D*_*intra*_ (and S/V) in the Mitra limit using the universal formula valid for oscillating gradients (Novikov and Kiselev, 2011), considering the nice linear trend when plotting ADC as a function of *f* ^−1/2^, which yields *D*_*intra*_ ~ 0.3 μm^2^/ms for NAA and tCho, and ~0.35 for tCr (and S/V from 2.4 to 2.9 μm^−1^). This seems relatively low, and compatible with the absence of short-range tortuosity when comparing to ADC values at longer *t*_*d*_ [ADC(*t*_*d*_ ~ 200 ms)~*D*_*intra*_/3] but simulations actually show that the Mitra regime is not strictly reached yet in this frequency range, and that the estimation of *D*_*intra*_ (but not S/V) remains biased toward lower values if spines/leaflets are present (Palombo et al., 2017).

Exploring the uncharted territory from ~700 to ~2,500 Hz to reliably determine *D*_*intra*_ and the intracellular tortuosity would require extremely powerful gradients. To reach *b* = 1 ms/μm^2^ at *f* = 2,500 Hz using a trapezoidal cosine waveform (Van et al., 2014) to maximize the weighting, while imposing total gradient waveform duration to be no more than 50 ms to retain some macromolecule signal (which is critical to discard datasets corrupted by motion artifacts; Ligneul and Valette, 2017), one would need a gradient coil capable of reaching ~5.5 T/m within 100 μs along each axis. This appears beyond reach in Humans, and very difficult to achieve in preclinical systems. Hence, the possibility to measure the intracellular viscosity in a “model-free manner” essentially depends upon uncertain technological breakthrough, and may remain elusive.

Although going to *t*_*d*_ ~ 2 s seems to already yield some sensitivity to long-range structure (in particular fiber length), this sensitivity remains relatively poor. Indeed, the observed ADC time-dependency at long *t*_*d*_, or the different time-dependency between neuronal and astrocytic metabolites, is quite close to ADC standard deviation, requiring averaging over many experiments. Further increase of *t*_*d*_ would allow enhancing the “ADC contrast” (relative decrease of ADC), which would subsequently lead to more reliable modeling and parameter estimation. For example, going to *t*_*d*_ ~ 10 s would approximately double the ADC contrast, for most situations with reasonable fiber length and complexity (see Figure 2 in Valette, 2014). Furthermore, going to such long *t*_*d*_ would help assessing the importance of phenomena that could potentially become significant at long time-scales (such as chemical exchange, intercellular exchange, active transport…) and that we have neglected so far, since structural effects alone appeared to satisfactorily explain data in the observed time-window.

Is it possible to reach *t*_*d*_ ~ 10 s (or longer)? Diffusion time is obviously limited by relaxation, and it is extremely difficult to measure diffusion much beyond metabolite T1. Increasing the magnetic field is a way to increase T1, but this gain becomes modest after 11.7 T (Lopez-Kolkovsky et al., 2016), therefore no significant jump beyond *t*_*d*_ ~ 2 s can be expected when increasing the magnetic field. However, it might be possible to observe metabolites with longer relaxation times under special conditions. For example, the ^13^C nuclei of the carboxyl groups of glutamate and glutamine have very long T1 (~10 vs. 1.5 s for ^1^H at high fields), due to the absence of strong dipole-dipole interaction, as these carboxyl groups share no chemical bond with proton. ^13^C natural abundance is too low (1.1%) to allow reliable detection in a reasonable time, however glutamate/glutamine can be labeled with ^13^C at the C5 carboxyl group, by intravenously infusing glucose labeled at position C2 and/or C5 (Sibson et al., 2001). In that context, signal might be detectable by direct ^13^C MRS. We actually tried to implement such an approach in the rat brain but, although some glutamate C5 signal could be detected, it remained too low for reliable ADC quantification. However, the progresses in radiofrequency coil technologies, and in particular the introduction of ^13^C cryogenic probes, might make this strategy viable in a near future. Another possibility might reside in the “long-lived states” (Levitt, 2010) where the dipole-dipole coupling is made ineffective (for systems with only two coupled spins-1/2, this corresponds to the antisymmetric “singlet state” with total spin *I* = 0). Some molecules with a high enough degree of symmetry can be brought to (using dedicated pulse sequences exploiting J-coupling), and then maintained in such states (using spin-locking), during a time significantly exceeding T1, thus allowing diffusion measurements over longer time-scales. This approach was already suggested for slowly diffusing compounds (Cavadini and Vasos, 2008), where the gradient strength can be limiting to induce enough diffusion attenuation, which can instead be increased by increasing *t*_*d*_. It is actually possible to bring some endogenous metabolites in such long-lived states. For example, taurine was shown to have long-lived state lifetime ~3 times longer than T1 (~2.7 vs. 1 s at 800 MHz; Ahuja et al., 2009). The extent to which long-lived state metabolite diffusion at ultra-long *t*_*d*_ may be measured *in vivo* remains to be explored.

## Conclusion

Over the last years, the range of DW-MRS diffusion times has considerably increased, now spanning approximately four orders of magnitude in the rodent brain (from ~0.2 ms to ~2 s). These measurements concurred with the vision that intracellular metabolites are neither massively transported by active mechanisms nor massively restricted in subcellular regions, but are primarily diffusing in long fibers, and that these fibers have some short-range and long-range structures (dendritic spines and astrocytic leaflets, fiber embranchments, finite fiber length…) that may also influence ADC time-dependency. The full elucidation—and accurate quantification—of these deviations from the simple infinite cylinders model requires increasing even further the range of *t*_*d*_ by at least one order of magnitude (e.g., from 0.1 ms to 10 s), if not more, which appears extremely challenging, yet not absolutely impossible.

## Author contributions

JV: Designed research and wrote the paper; CL, CM, CN: Performed most of the experiments described in this article and proofread the paper; MP: Performed most of the modeling described in this article and proofread the paper.

### Conflict of interest statement

The authors declare that the research was conducted in the absence of any commercial or financial relationships that could be construed as a potential conflict of interest. The handling editor is currently editing a Research Topic with one of the authors JV, and confirms the absence of any other collaboration.
